# Generation Length: Often Ignored and Sometimes Misunderstood

**DOI:** 10.1111/eva.70256

**Published:** 2026-07-09

**Authors:** Fred W. Allendorf, Nils Ryman

**Affiliations:** ^1^ Division of Biological Sciences University of Montana Missoula Montana USA; ^2^ Department of Zoology Stockholm University Stockholm Sweden

**Keywords:** age at first reproduction, divergence time, effective population size, generation length, historical demography, life history

## Abstract

Traditional mathematical models of population genetics have generally assumed discrete generations, but most species have overlapping generations. It is necessary to estimate generation length with overlapping generations for purposes of comparison with discrete‐generation models. In populations with overlapping generations, generation length is the average age of parents at the time their progeny are born. Genetic risks associated with small populations depend on both effective population size and elapsed time in generations. In some cases, populations with larger effective size can lose heterozygosity more quickly than populations with smaller effective size because they have shorter generation length. Therefore, estimates of effective size alone cannot be used to predict the loss of heterozygosity over calendar time. Generation length is also required to estimate effective population size from the rate of loss of heterozygosity, to estimate the time of divergence between species using molecular data, and to use coalescent models for historical demographic analyses. Generation length is often not carefully defined or estimated in these cases. In some papers, authors have used the generation length only for females and have ignored males. A review of the literature indicates that the generation length for males and females can be substantially different in some species. In addition, a variety of proxies have been used in the literature for generation length. Many of these proxies do not provide reliable estimates of generation length. Accurate estimates of generation length require detailed life‐history information that is unfortunately not available for many species. Analyses using these proxies for generation length should be treated with some skepticism. Most population genetics textbooks do not define generation length. It is hard to understand why such an important parameter in population genetics has been so ignored.

## 1 Introduction

Traditional mathematical models of population genetics generally assume discrete generations in which a generation is fully replaced by the next before reproduction happens again (Ryman 1997). However, most species (perhaps 90%) have overlapping generations in which individuals born at different times can reproduce at the same time. For example, annual plants are one of the primary groups said to have discrete generations, but many annual plants have soil seed banks, so that they have overlapping generations. There is no such thing as a “generation” in populations with overlapping generations. Nevertheless, it is necessary to define generation length (*G*) with overlapping generations for purposes of comparison with discrete‐population models.

Overlapping‐generation models are far more complex than discrete‐generation models; this is primarily because Hardy–Weinberg proportions cannot be assumed with overlapping‐generation models (p. 15, Felsenstein 2019). Discrete‐generation populations will be in Hardy–Weinberg proportions after one generation of random mating. In contrast, overlapping‐generation populations will approach Hardy–Weinberg proportions asymptotically over many generations.

Generation length is a crucial parameter in the application of genetics to evolution and conservation. For example, genetic risks associated with small populations depend primarily on effective population size (*N*_e_) and elapsed time in generations. Generation length is required to use inbreeding *N*_e_ to estimate the loss of heterozygosity over calendar time. Generation length is also required to estimate times of species divergence and to reconstruct demographic history from molecular data. In addition, generation length is crucial for assessing population declines. For example, the International Union for Conservation of Nature (IUCN) uses the rate of decline in abundance per generation to assess threat status (IUCN 2024).

The purpose of this paper was to review the literature on the estimation and use of generation length in population and conservation genetics. The first objective of this paper was to clarify the definition of generation length. The second objective was to explain the importance of using generation length to understand the genetics of populations. Finally, we present common misunderstandings of generation length in the literature and consider their implications.

## 2 What Is Generation Length?

Generation length is crucial for converting the rates of genetic evolution to calendar time in species with overlapping generations. For example, estimates of generation length in populations with overlapping generations are required for predicting the loss of heterozygosity over calendar time because heterozygosity is lost at a rate of 1/2*N*_e_ per generation, where *N*_e_ is the inbreeding effective population size.

In his classic 1931 paper, Wright demonstrated that heterozygosity would decline by 1/2 *N* per generation, where *N* is the population size in an ideal population. He also presented several situations in which the “effective” number is smaller than *N*: fluctuating population size, unequal number of males and females, and nonrandom variation in reproductive success. Wright (1938) introduced the concept of effective population size and presented formulas, which corrected for these effects to estimate the effective number (*N*_e_).

Felsenstein (1971) presented a haploid model with fixed numbers in each age class that described the generational effective size in a population with overlapping generations. It is surprising that Wright's (1931, 1938) discrete‐generation model describing the loss of heterozygosity due to genetic drift was not extended to overlapping generations until several decades later. Felsenstein (1971) demonstrated that generation length is the average age of parents at the time their progeny are born. Hill (1972, 1979) developed a more general model with overlapping generations, and he showed that the effective number is the same as that for discrete‐generation populations, where the generation length is the mean age of parents at the birth of their progeny. That is, generation length is the weighted mean parental age at reproduction, where the weighting considers the number of offspring produced at each age.

## 3 Importance of Generation Length

### 3.1 Predicting Loss of Heterozygosity and Estimating *N*_e_

Estimating *N*_e_ has become a common practice in describing the genetics of populations in order to describe the loss of genetic variation. However, it is necessary also to estimate *G* to predict the loss of heterozygosity over calendar time. This is especially important when comparing the effects of different management schemes on the rate of loss of heterozygosity because conditions that increase *N*_e_ often decrease the generation interval.

In fact, population size and generation length are inversely related between species (Chao and Carr 1993; Lewin and Eyre‐Walker 2026). This relationship played a prominent role in the development of the neutral theory of molecular evolution because it was used to explain why a molecular clock is compatible with the theory (Kimura 1983; Ohta and Tachida 1990).

**FIGURE 1:**
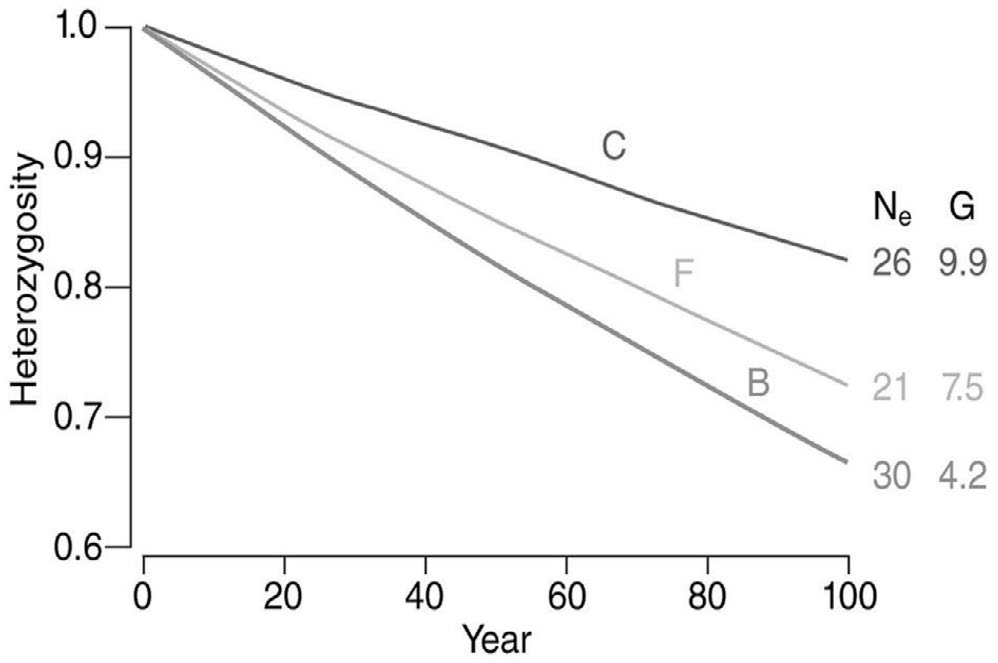
Expected decline in heterozygosity under three different sets of hunting regulations in moose from Sweden with a census size of 100 adults following the hunting season. The effective population size (*N*_e_) and generation length (*G*) for each hunting regime are indicated on the right. In hunting regime B, all adults experience identical mortality rates, but calves (less than 1 year old) are protected and not hunted. In C, only calves are hunted. In F, adult females with calves are protected so that the risk of mortality of an adult female is reduced as a function of the number of calves (0, 1, or 2) with her at the beginning of the hunting season. The regime (B) with the largest *N*_e_ is expected to lose heterozygosity at nearly twice the rate of the regime (C) with a smaller *N*_e_ that has a longer generation interval. Redrawn from Ryman et al. (1981).

Population size and generation length also tend to be inversely correlated among populations within species. For example, Ryman et al. (1981) found that different hunting regimes for moose ( *Alces alces* ) in Sweden have strong effects on both *N*_e_ and *G*. Regimes in which much of the reproduction resulted from younger animals resulted in the largest *N*_e_ because more animals reproduced (e.g., Figure 1, line B). However, these regimes also had shorter generation intervals because young animals produced most of the progeny. In contrast, regimes in which most of the reproduction resulted from fewer older animals (e.g., line C in Figure 1) resulted in smaller *N*_e_ with longer generation lengths. Thus, populations with smaller *N*_e_ sometimes lost heterozygosity at a slower rate over calendar time because those effects of hunting resulted in longer generation time that reduced *N*_e_ (Figure 1).

Tanaka et al. (2009) considered different management strategies to best maintain genetic variation in overabundant koala ( *Phascolarctos cinereus* ) populations that have low genetic variation. They considered the effects of alternative management plans on *N*_e_. However, they did not consider generation interval. It is possible that some strategies producing larger values of *N*_e_ might actually lose heterozygosity at a faster rate over calendar time than strategies resulting in smaller values of *N*_e_, as in the moose example. In fact, the strategy recommended to increase *N*_e_ was to administer contraception to all females beyond a particular age; this strategy would reduce the generation interval and increase the rate of loss of heterozygosity over calendar time.

Hard et al. (2006) modeled the effects of hunting on effective population size and generation length in European red deer (*Elaphus elaphus*) and North American wapiti ( *E. canadensis* ). They found that *N*_e_ generally increased with increasing generation length. Unfortunately, they did not use the standard definition of generation length. They defined generation length as the mean age at reproduction. This value will be the same as the average age of parents at the birth of their progeny only if reproduction was uniform throughout the reproductive life span. It is difficult to evaluate the results of Hard et al. (2006) because they do not consider how using the standard definition would have affected their results (e.g., over‐ or underestimate or different standard errors).

Nunney (1993) explored the effects of the mating system on the relationship between effective population size and generation interval. He considered both monogamy and harem polygyny. In all of the monogamous cases that he considered, lengthening the generation time reduced the effective population size. In highly polygynous mating systems, effective population size generally increased with generation time because the variance in male reproductive success decreased with increasing generation time.

Generation length is also required to estimate inbreeding *N*_e_ based on the loss of heterozygosity over time. *N*_e_ can be estimated from the observed rate of loss of heterozygosity and the generation length because heterozygosity is lost at a rate of 1/2*N*_e_ per generation. The rate of loss of heterozygosity and *G* can be calculated in simulation models (e.g., Lacy and Pollak 2026). These values can then be used to estimate *N*_e_ (Harris and Allendorf 1989). This method also can be used to estimate *N*_e_ with empirical data (Hauser et al. 2002).

### 3.2 Estimating Times of Divergence and Reconstruction of Demographic History

The time since two lineages shared a common ancestor (split time) can be estimated by measuring the amount of sequence divergence. Converting the amount of sequence divergence to split time requires knowing the mutation rate and generation length. Split times of 4–6 million years between humans and the great apes based upon sequence divergence are generally accepted (Hobolth et al. 2007; Levinstein Hallak and Rosset 2024). However, these split times are based upon using generation lengths for chimpanzees ( *Pan troglodytes* ) and mountain gorillas ( *Gorilla beringei* ) that have no apparent empirical basis (Langergraber et al. 2012). These divergence times are in conflict with split times based upon the fossil record.

Langergraber et al. (2012) used direct genetic parentage analysis to estimate the average age of parents for females and males in chimpanzees and mountain gorillas. Their estimates of generation length of 24.6 in chimpanzees and 19.3 in mountain gorillas are larger than those of generation times assumed in previous studies. They estimated the human–chimpanzee split to be approximately 7–8 million years ago and the human gorilla split to be 10–11 million years. These times are in agreement with split times based upon the fossil record.

Generation time is also crucial when using coalescent models for historical demographic analyses (Kuhner 2009). Genetically based demographic reconstructions use contemporary patterns of genomic variation and the mutation rate to infer the past trajectory of *N*_e_. Despite its importance, *G* is often not carefully defined or estimated when used in genetically based demographic reconstructions (see Bakker et al. 2022).

Robinson et al. (2021) used Markovian coalescent methods to reconstruct the historical population sizes of California condors ( *Gymnogyps californianus* ) and turkey vultures ( *Cathartes aura* ) using chromosome‐length genome assemblies. These authors conclude that California condors were more abundant than turkey vultures for much of the Pleistocene and that understanding historical patterns of population size is useful for current management.

Bakker et al. (2022) questioned the findings of this study because the authors used the same value (10 years) for *G* in these species even though they have vastly different life histories. For example, the age of first reproduction is 10 and 3 in California condors and turkey vultures, respectively. The estimated life span (maximum observed longevity) is 60 and 17 in California condors and turkey vultures, respectively. Robinson et al. (2022) estimated that *G* is 25 and 7 in California condors and turkey vultures, respectively. This shorter generation time inflates estimates of historical effective population size and makes the relative abundance comparisons flawed (Bakker et al. 2022). See Robinson et al. (2022) for a response to these criticisms. Regardless of which of these groups of authors is correct, it is inappropriate that Robinson et al. (2021) used the same *G* values for species with such vastly different life histories.

### 3.3 Evaluating Population Declines

The IUCN considers five criteria in assessing extinction risk to assign a Red List risk category (from least concern to extinct) for a species (IUCN 2024). Generation length is used in all of the five criteria for the temporal scaling of population declines. For example, reductions under Criterion A are considered over 10 years or three generations (whichever is longer, but up to a maximum of 100 years for future reductions). For criteria B and D, a short time frame is defined as one within a single generation or 3 years, whichever is longer. Criterion C, which considers population size, structure, and trends, is scaled by generation length.

Therefore, it is important to have an accurate estimate of generation length. Over‐ or underestimates of *G* cause the decline to be measured over the wrong time period, which leads to an over‐ or underestimate of extinction risk. Inclusion of a generation‐length metric is important because *G* is a strong correlate of extinction risk (O'Grady 2008).

Bird et al. (2020) estimated the generation length for all bird species and considered their implications for extinction risk. The bird generation lengths ranged from 1.42 to 27.87 years, with a median of 2.99 years. They found that most species (61%) have generation lengths shorter than 3.33 years. Therefore, the period of three generations is shorter than 10 years, the value used by Criterion A of the IUCN Red List. They conclude that extinction risks are best evaluated by scaling population trends by generation lengths.

## 4 Common Misunderstandings

This review was motivated by many papers in the literature that demonstrate a misunderstanding of generation length.

### 4.1 Differences Between Males and Females

Some demographic definitions of generation length that focus on population dynamics only use females (e.g., p. 501, Charlesworth and Charlesworth 2012). Nevertheless, the genetic generation length needed for predicting the loss of heterozygosity and phylogenetic inference requires consideration of both sexes. Jonasson et al. (2022) presented a “unifying framework” for estimating generation time for use in predicting the loss of heterozygosity and making phylogenetic inference. However, all three estimates of *G* that they present are based only on mothers, with no consideration of fathers. Similarly, Staerk et al. (2019) considered the performance of estimators of generation time used for assessing extinction risk with the IUCN criteria and for estimating evolutionary rates. However, all of their estimators of *G* are based only on mothers, with no consideration of fathers.

Differences in generation length between females and males can be substantial (Table 1). For example, in humans *G* for fathers is 32% longer than *G* for mothers. The opposite is true for brown bears; *G* for mothers is 33% longer than *G* for fathers.

**TABLE 1:** Generation length and the proportional difference between females and males. Human values from Wang et al. (2023); chimpanzee and mountain gorilla values from Langergraber et al. (2012); all other values personal communication from R.S. Waples based on Waples et al. (2013).

Common name	Scientific name	Female	Male	Mean	Difference
Human	*Homo sapiens*	23.20	30.70	26.95	−32.3%
American bison	*Bison bison*	6.44	8.31	7.38	−29.1%
Great tit	*Parus major*	1.73	2.00	1.86	−15.1%
American bull frog	*Rana catesbeiana*	5.03	5.20	5.11	−3.3%
Mountain gorilla	*Gorilla beringei*	18.19	20.37	19.28	−1.2%
Reed deer	*Cervus elaphus*	8.46	8.32	8.39	1.7%
Mountain sheep	*Ovis canadensis*	5.80	5.68	5.74	2.1%
Cascade frog	*Rana cascadae*	3.36	3.28	3.32	2.3%
Chimpanzee	*Pan troglodytes*	25.18	24.08	24.63	4.4%
Mole crab	*Emerita talpoida*	1.09	1.04	1.06	4.6%
Eurasian sparrowhawk	*Accipiter nisus*	8.82	8.17	8.49	7.4%
Bottlenose dolphin	*Tursiops truncatus*	15.55	13.87	14.71	10.8%
African lion	*Panthera leo*	7.00	6.22	6.61	11.1%
Brown trout	*Salmo trutta*	8.73	7.52	8.13	13.8%
Belding's ground squirrel	*Spermophilus beldingi*	3.34	2.84	3.09	14.9%
Red drum	*Sciaenops ocellatus*	12.54	10.14	11.34	19.1%
Brown bear	*Ursus arctos*	12.61	8.42	10.52	33.2%

If *G* differs between the sexes, the generation length is simply the mean *G* for mothers and fathers. The IUCN (2024) guidelines for dealing with differences between mothers and fathers are incorrect:

If the estimate of generation length differs between males and females it should be calculated as a weighted average, with the weighting equal to the relative frequency of reproducing individuals of the two sexes.

Males and females each contribute one‐half of the genes to the next generation. Therefore, the male and female generation times should be averaged without weighting.

### 4.2 Age of First Reproduction

Many papers in the literature have used the age of first reproduction (*F*), or twice this age (2*F*), as the generation length. There appear to be different motivations for using each of these two values. *F* appears to be sometimes used because the authors misunderstand the definition of generation length (Bowie et al. 2006; Padró et al. 2020; Madsen et al. 2023). In contrast, some papers have used 2*F* as a proxy for *G*.

We contacted Madsen about the use of the age of first reproduction (*F*) for *G* in his study of a bottleneck in an isolated island population of adders ( *Vipera berus* ; Madsen et al. 2023). In this case, 80% of the males mature at 3 years, and 80% of the females mature at 4 years (T. Madsen, personal communication). The paper uses a generation length of 3 years. However, according to Madsen (personal communication), the average age of fathers is about 5 years and the average age of mothers is about 6 years so that *G* should be about 5.5 years, not 3 years.

**FIGURE 2:**
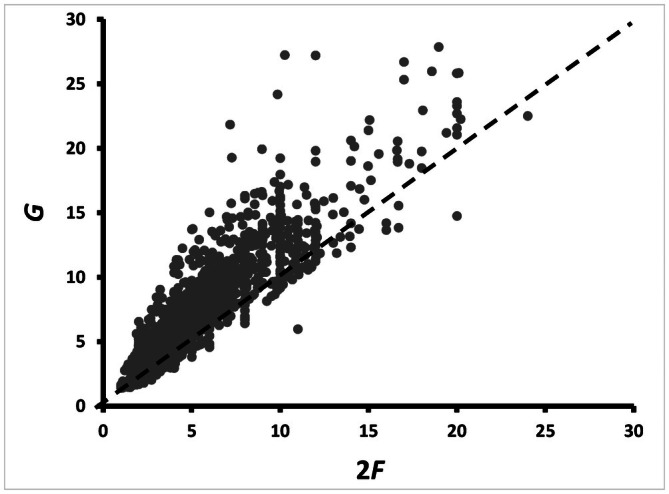
G**e**neration length (*G*) as a function of twice the age of first reproduction (2*F*) for 1050 species of birds. Data from Bird et al. (2020).

Other papers have used 2*F* as an approximation for generation length without providing any empirical justification (see Bakker et al. 2022). For example, Nadachowska‐Brzyska et al. (2015) used 2*F* as a proxy for *G* in their study of the temporal dynamics of 38 bird species during the Pleistocene. Bird et al. (2020) compiled published life‐history data and estimated *G* for all bird species. Generation lengths were derived based on age at first breeding, maximum longevity, and annual adult survival (Figure 2). *G* is greater than 2*F* in 93% of all bird species.

The IUCN recommends two proxies for *G* that are based on age at first reproduction, reproductive life span, and adult mortality rate. Fung and Waples (2017) evaluated the performance of these proxies for *G*. They found large mean errors for all proxies and concluded that it is necessary to have detailed life‐history information to calculate reliable estimates of generation length. We agree with these authors; accurate estimates of *G* require detailed life‐history information that is unfortunately rarely available for many species. Analyses using these proxies for *G* should be treated with skepticism. If reliable life‐history information is not available for a species, we suggest performing analysis with different values to determine how sensitive the results are to different values of *G*.

## 5 Summary and Recommendations

Generation length is a crucial parameter that is used in a variety of applications of population genetic data. We were surprised to discover during our review of the literature that *G* is often ignored, incorrectly defined, or incorrectly estimated. In addition, most population genetics textbooks do not define generation length. It is hard to understand why such an important parameter in population genetics has been so ignored.

We make the following three recommendations:

1. All estimates of *N*_e_ used to predict the loss of heterozygosity over calendar time should be accompanied by estimates of *G*.
2. All genetic estimates of times of divergence and coalescent reconstructions of demographic history should include an explanation of how *G* was estimated.
3. If reliable life‐history information is not available for a species, we suggest performing analysis with different values to determine how sensitive the results are to different values of *G*.

## Funding

N. Ryman was supported by grants to Linda Laikre from the Swedish Research Council (VR 2019‐05503 and VR 2025‐04811).

## Conflicts of Interest

The authors declare no conflicts of interest.

## Data Availability

Data sharing not applicable to this article as no datasets were generated or analysed during the current study.
